# Reducing disparities in mammography-use in a multicultural population in Israel

**DOI:** 10.1186/1475-9276-8-19

**Published:** 2009-05-19

**Authors:** Orna Baron-Epel, Nurit Friedman, Omri Lernau

**Affiliations:** 1 School of Public Health, Faculty of Social Welfare and Health Sciences, University of Haifa, Haifa, Israel; 2 Research and Evaluation Department, Maccabi Healthcare Services, Israel; 3 Department of Epidemiology and Preventive Medicine, School of Public Health, Sackler Medical Faculty, University of Tel Aviv, Tel Aviv, Israel; 4 Faculty of Medicine, Ben Gurion University, Bear Sheba, Israel

## Abstract

**Background:**

In the past mammography-use has been reported to be low in Israel compared to other western countries. The objectives of this study were (1) to assess the increase in mammography-use during the years 2002 to 2007 in four population groups in Maccabi Healthcare Services (MHS), Israel: non-immigrant non-ultraorthodox, ultraorthodox, and immigrant Jewish women and Arab women; (2) to assess ethnic and socioeconomic disparities in mammography-use.

**Methods:**

A random telephone survey of 1,550 women receiving healthcare services from MHS was performed during May-June 2007. Information from MHS claims-records database regarding mammography-use was obtained for each woman for the period 2002 to 2007. Since mammography-use serves as a quality assurance measure for primary care, MHS sent mail and telephone invitations for mammography to all women since the end of 2004.

**Results:**

At the beginning of the follow-up period (2002) mammography-use among Jewish non-immigrant non-ultraorthodox and ultraorthodox women was higher than among Arab and Jewish immigrant women. During the 5 year follow-up these disparities decreased significantly. In 2007, mammography-use by Arab women was only slightly lower compared to all groups of Jewish women. In 2007, after adjustment for socioeconomic factors there was only a borderline significant difference between Jewish and Arab women. The socioeconomic variables were not associated with mammography-use in 2002 and 2007 in any of the groups except for marital status in immigrant women in 2002.

**Conclusion:**

The interventions implemented by MHS may have increased mammography-use in all population groups, decreasing disparities between the groups, however the differences between Jewish and Arab women have not been completely eliminated and indicate a need for further targeted interventions. No significant socioeconomic disparities in mammography-use were observed.

## Introduction

Breast cancer in Israel is the most common cancer among women [1]. The rate of breast cancer in Jewish women is similar to the rate among women in the USA and other western countries such as the Netherlands and Canada [2,3]. In 2002, the age adjusted incidence rate of breast cancer in Israel among Jewish women was more than twice that of Arab women [1]. The incidence rate among Arab women in Israel is similar to that found in neighboring countries such as Jordan [2]. Among both Arab and Jewish-Israeli women, the incidence rates have increased during the last decade. This increase is especially pronounced among Arab women whose age-adjusted incidence rate increased by 202% from 1979–1981 to 2000–2002, while among Jewish women, the rate increased by 46%, during the corresponding years [4]. In addition it seems that Arab women are more frequently diagnosed with advanced stages of breast cancer compared to Jewish women [4]. This corresponds with similar results in the USA, where minority populations such as Blacks and American Indian women were diagnosed at later stages of breast cancer compared to non-Hispanic white women [5,6]. This later stage of diagnosis may depend on lower frequency and rates of mammography screening [6,7].

Screening for breast cancer using mammography was shown to effectively reduce mortality from breast cancer in women aged 50–74 [8,9]. Therefore, the major medical organizations recommend mammography for women [10,11]. The recommendations in Israel are that women aged 50–74 have a mammogram every two years, and women at high risk (family history) have a mammogram every year, from the age of 40.

Many studies have found disparities in the rates of mammography-use between ethnic/racial population groups. In the past, recent immigrant, Black, Asian and Hispanic women in the USA were found to adhere less to screening mammography [12-15]. Most of these disparities can be explained by lower socioeconomic status and lack of health insurance [12,13]. Low income was a predictor of low mammography-use more so than education or race [16]. Some of the disparities were suggested to be due to inequalities in access to healthcare; studies showed that when financial barriers were removed mammography-use increased, but only to a limited extent [17,18].

However, the availability of the service with no financial barrier is not enough to guarantee high level of compliance with screening. Many other barriers to mammography-use have been identified, such as various attitudes and beliefs (perceived risk, fatalism, normative beliefs and more) as well as technical barriers (distance from clinic, gender of technician and more) [19].

In the USA there is an increase in the use of screening mammography with increased rates in every demographic group. However, disparities still persist among ethnic/racial minorities and low-income women [20]. Since the year 2000 no further increase has been observed in women over 40 and maybe even a small decrease in mammography-use has occurred [16].

Programs increasing the adherence of women with recommended mammography have been implemented and proven to be effective. Interventions include various strategies, such as, chart-based interventions to remind physicians to refer for a mammogram, reminder-based interventions including mail or phone reminders, interventions addressing financial or logistic barriers, and culturally tailored interventions aimed at lowering specific barriers [9,21-23].

In Israel, there are four major culturally distinct populations [24]: 1) Non-immigrants, non-ultraorthodox Jewish women; this group includes the majority of women in Israel-around 60% of the female population. 2) Ultraorthodox Jewish women, a group that is distinguished by regional isolation and relative homogeneity in their communities and specific characteristics such as high fertility. This group is estimated to be about 6–8% of the Israeli female Jewish population. 3) Immigrant Jewish women, who arrived in Israel from the former Soviet Union (fSU) during the last 19 years and who have a distinct cultural background and language (about 17% of the female population in Israel). 4) Arab woman, having their distinct religion, culture and language (Arabs are 19% of the population in Israel). Other smaller groups exist such as immigrants from other countries [24].

There is evidence of large differences in mammography-use between the population groups. The age adjusted rate of self-reported mammography-use among Arab women was much lower compared to Jewish women. In 2003–4, self-reported mammography-use during the preceding two years, in the general population, was 71% among all Jewish women and 47% among Arab women (age 50–74) [25]. Another study estimated the rate of mammography-use among Arab women aged 50–74 as only 20% [26]. Immigrant women from the former Soviet Union (fSU) also reported lower levels of mammography-use compared to non-immigrant Jewish women. In the same survey run during 2003–4, 59.8% of immigrant women (aged 50–74) arriving in Israel during the years 1990–1998 from the fSU reported having a mammogram during the past two years. This number was even lower for immigrant women arriving in Israel from the fSU during the years 1999–2004 (49.3%), compared to 71% among non-immigrant Jewish women living in Israel from before 1989 [27]. No published data on mammography-use is available for ultraorthodox women.

In Israel, a National Health Insurance Law provides healthcare services to all citizens including immigrants. The basket of services covered by the National Health Insurance Law includes a biennial mammogram for women aged 50–74 and an annual mammogram for women above 40 at high risk.

As part of the National Health Insurance every citizen is entitled to receive healthcare from one of four health care service organizations. Maccabi Healthcare Services (MHS) is the second largest healthcare service in Israel and serves over 1.7 million members, about 25% of the Israeli population. The enrollees in MHS do not represent the total Israeli population; however, they do include all population groups composing the Israeli population. Although since 1998 MHS has sent out letters of invitation for mammography to women age 50–74, this effort was somewhat sporadic. Since the end of 2004 MHS has put high priority into increasing levels of adherence with mammography recommendations in all population groups as rates of mammography-use serve as a quality assurance measure for primary healthcare services. Letters of invitation for mammography were sent out regularly to women age 50–74 who had not had a mammogram in the previous two years; these letters were sent out in Hebrew, Russian and Arabic to the corresponding population groups. The local clinics' staff made telephone calls to all women not having had a mammogram during the previous two years in order to remind them to have a mammogram. In most cases a mammogram is scheduled during this phone call. In addition, a mobile mammogram unit was sent out to periphery towns in order to decrease access problems. The mobile mammogram units performed 4000 mammograms during 2006, this consisted of 5% of all screening mammograms funded by MHS that year. Therefore it was appropriate to measure the rates of mammography before and after the implementation of this quality assurance measure.

The objectives of this study were to assess the increase in mammography-use during the years 2002 to 2007 in four population groups in Maccabi Healthcare Services, (MHS): non-immigrant non ultraorthodox-, ultraorthodox-, and immigrant-Jewish women and Arab women. In addition, the objective was also to asses ethnic and socioeconomic disparities in mammography-use, at the beginning and end of this follow-up period.

This information may help in assessing the disparities in mammography-use in the population and in identifying groups for which disparities still exist. The gold standard for evaluation of interventions is the randomized control trial; however, since it is not possible ethically to have a control group at this stage of knowledge regarding mammography, analysis of the population receiving the services can give an idea regarding the success of the intervention run by MHS and give ideas for future needs of each population.

## Methods

### Population sample

Maccabi Healthcare Service (MHS) is a large Health Maintenance Organization (HMO) that insures about 25% of the Israeli population. Four random samples of women aged 52–74 were obtained from MHS computerized list of members after identifying the four groups in the database. This age group was chosen so as to make sure the women were all entitled to have a mammogram during the previous two years, as recommended. Women reporting having been diagnosed with breast cancer were excluded from the sample (75 women). The four groups consisted of: 1. Non-ultraorthodox Jewish women living in Israel from before 1989, (referred to as: non-immigrant, non-ultraorthodox Jewish women) 2. Women defined as ultraorthodox. This definition was determined by the MHS, in their capacity to plan culturally sensitive medical services; this group did not include immigrants which arrived after 1989 from the fSU, 3. Immigrant women arriving in Israel since 1989 from the fSU (referred to as immigrant women), and 4. Arab women living in Arab villages and towns but not in mixed towns. This group includes over 90% of Arab women. Only 10% of Arabs live in mixed towns. Immigrants from other countries were not included in the study and orthodox or religious women not from the fSU were included in group1. The criteria for calculating the sample size was the rate of mammography-use among Arab and Jewish women during the last 2 years in 2003–4 (estimated at about 50% of Jewish women and 30% of Arab women). A sample size of at least 300 women in each group was needed to identify significant differences between the groups. The list included the woman's name and home phone number.

The study was approved by the MHS's ethical committee.

### Data collection and questionnaire

The questionnaire was administered over the telephone by trained female interviewers from the corresponding population group for each language, Hebrew, Arabic, and Russian. The interview took about 30 minutes. Arab women interviewed Arab women and Russian speaking women (immigrants from fSU) interviewed the immigrants from the fSU. The non-immigrant, non-Arab women were interviewed in Hebrew. Quality control of the interviewing phase included regular training of the interviewers, interview simulations, and interviewing under direct observation. The survey was performed during the months May and June 2007.

**Figure 1:**
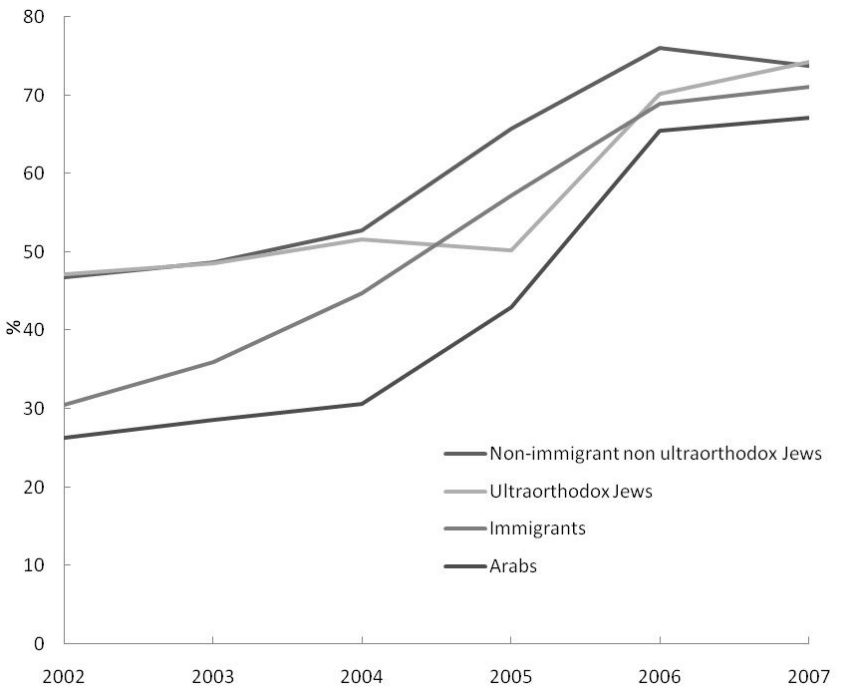
**Frequency of mammography-use during the previous two years by year and population group (percent)**.

The response rate was 71.5%; 1,550 women were interviewed from a sample of 2169; 619 refused to be interviewed. The interviewed groups included 399 non-immigrant, non-ultraorthodox Jewish women, 385 ultraorthodox Jewish women, 384 immigrants and 392 Arab women. The response rates were 69.3%, 56.0%, 77.1% and 92.9% respectively. Fourteen women did not finish the whole questionnaire, these women were included in figure 1 but not in the regressions. Missing data on specific questions reduced the sample size for the regressions.

The questionnaire included questions regarding socioeconomic status, mammography-use during the previous two years, attitudes and beliefs towards breast cancer in the past. The questionnaire was translated into Arabic and Russian and translated back into Hebrew to ensure correct translation. A pretest of 50 women was performed before running the full scale survey.

Members of MHS eligible for mammography have the option of receiving mammography from the MHS radiology clinics or from participating providers. In all cases in which the mammography service is reimbursed by MHS, regardless of provider, the MHS automated claims system registers the service. The dates of mammography performance for each woman answering the survey were added to the survey data. All mammograms performed since 2000 up to the time of the interviews were recorded. According to an internal MHS survey only 1% of women age 50–75 had a mammogram they paid for themselves, thus resulting in it not being registered in the claims records.

### Study variables

The four population groups were defined by the data from MHS and reassessed by self-reported questions regarding religiosity, ethnic background, from were the woman immigrated and year of immigration. About 20 women in the ultraorthodox group reported themselves as not being ultraorthodox and were transferred to the non-immigrant, non-ultraorthodox group. Immigrant women not from the fSU were not included in the study

Women having had at least one mammogram up to two years prior to the date of the interview, as registered in the claims database, were categorized as having had a mammogram according to claims records during the two years previous to 2007. The exact date of the mammogram was extracted from the claims database. Not having a mammogram during the last two years was categorized as 0 and having a mammogram was categorized as 1. In this way, mammography-use was calculated for each year from 2002 to 2007. Only women that were entitled to a free mammogram during the previous two years at each point in time were assessed. For example, in 2002, only women aged 52 and over at the time (in 2002) were entered into the analysis. Therefore the sample for analysis of mammography-use in 2002 was smaller than the sample analyzed for the year 2007 as the sample was extracted by age in 2007.

All other variables were self-reported as MHS does not have socioeconomic status variables in the database. The interviewee indicated age. Thirty seven women would not give their age. Employment status was categorized as working (1) or not working out of the home (0) (unemployed, retired, housewife). Education was assessed by three categories: 12 years of schooling or less (1), a high school diploma and other schooling after high school (2) and receiving an academic degree (3). These two variables served as measures of socioeconomic status. Marital status was categorized as two groups, married or living with a spouse (1) and without a spouse (0), the latter being a combination of divorced, living separately, single, or widowed.

### Statistical analysis

Multivariable logistic regression analysis with mammography-use as the dependant variable was performed for the entire population adjusting for the socioeconomic variables available from the questionnaire; these were hypothesized to be associated with mammography-use as suggested in the literature. Population-group was added as a categorical variable, where non-immigrant non-ultraorthodox Jewish women were the reference group. Age was added as a continuous variable and the other variables were categorical variables. Further logistic regression models were run separately for each population group.

Statistical significance was set at a p value of less than 0.05, SPSS version 14.0 software was used for the analysis.

## Results

### Study population

The sample consisted of four population groups with a mean age of 60.1 (SD 6.1); the Arab women were younger (59.7) and the immigrants were older (61.8). A little less than half of the Jewish women worked out of the home (48.5%, 47.2% and 44.5% of non-immigrant non-ultraorthodox, ultraorthodox and immigrant women respectfully), among Arab women only 11.3% worked out of the home. Education varied between groups, the immigrants reported most frequently having an academic degree (66.8%), the Arab women least frequently (12.4%), 34.6% and 23.5% of the non-immigrant non-ultraorthodox Jewish women and ultraorthodox women reported having an academic degree. Most of the ultraorthodox women reported being married (90.2%) and only 59.8% of the immigrant women were married, Arab and non-immigrant non-ultraorthodox Jewish women had similar rates of being married (76.2% and 72.7% respectively). These differences between groups were significant for all variables.

### Increase in mammography-use

In 2002, 46.8% (130 women) of the non-immigrant, non-ultraorthodox Jewish women and 47.2% (119 women) of ultraorthodox women had a mammogram during the previous two years. However, only 30.5% (89 women) and 26.3% (66 women) of immigrant and Arab women respectively had a mammogram during the previous two years. Figure 1 presents the increase in mammography-use in the four population groups during the five years from 2002 to 2007. Most of the increase in use occurred between the years 2004 and 2006. Among the ultraorthodox, most of the increase occurred during the year 2005–2006. The increase in use among immigrant women occurred throughout the whole period, from 2002 to 2007.

In 2007, the non-immigrant, non-ultraorthodox Jewish women, ultraorthodox and immigrant women had similar rates of mammography-use, 73.8% (299 women), 74.3% (263 women) and 71.1% (273 women) respectively. The Arab women had a mammogram registered in the claims data less often (67.2%, 256 women). The difference in mammography-use between Arab and Jewish women was significant (p = 0.01). The absolute difference between Arab women and non-immigrant non-ultraorthodox Jewish women in 2007 was much smaller compared to the difference in 2002 (6.6% compared to 20.5%).

As the four population groups differed in their socioeconomic status multivariable logistic regression models were run for the years 2002 and 2007, to assess the differences between the groups after adjusting for the socioeconomic variables (Table 1). In 2002, the odds ratio (OR) for having a mammogram for both immigrant and Arab women was less than half, relative to the non-immigrant, non-ultraorthodox Jewish women (OR = 0.49, CI = 0.34–0.71 for immigrants and OR = 0.43, CI = 0.29–0.65 for Arab women), and there was no significant difference between non-immigrant, non-ultraorthodox Jewish women and ultraorthodox women (OR = 0.93, CI = 0.70–1.41). In 2007, there was no significant difference between the Jewish population groups. It seems that the OR for Arab women having a mammogram in 2007 was lower compared to Jewish women, although the p value was borderline (p = 0.053). Age, education, and employment were not associated with mammography-use for both years. In 2002, the OR for having a mammogram indicates that the odds of an unmarried woman having a mammogram were lower than a married woman; however this difference was not significant in 2007.

**Table 1:** Factors associated with mammography-use during the previous two years in 2007 and 2002.

	**2007** **N = 1,485**			**2002** **N = 1,057**		
	N	OR	(95% CI)	N	OR	(95% CI)

Population group						

Non-immigrant non-ultraorthodox	399	1.00	reference	273	1.00	reference

Ultraorthodox	347	0.99	(0.71, 1.39)	247	0.93	(0.70, 1.41)

Immigrants	374	0.92	(0.59, 1.15)	291	0.49*	(0.34, 0.71)

Arabs	365	0.71***	(0.51, 1.01)	246	0.43*	(0.29, 0.65)

						

**Age**	-	1.01	(0.99, 1.03)	-	0.99	(0.96, 1.01)

Education						

Academic	513	1.00	reference	361	1.00	reference

High school diploma	468	0.95	(0.71, 1.28)	333	0.83	(0.60, 1.15)

Less than High school diploma	504	0.82	(0.59, 1.15)	363	0.79	(0.55, 1.14)

Marital status						

Not married	382	1.00	reference	299	1.00	reference

Married	1103	1.20	(0.92, 1.57)	758	1.36**	(1.00, 1.85)

Employment						

Unemployed	918	1.00	reference	721	1.00	reference

Employed	567	0.88	(0.67, 1.16)	336	1.01	(0.75, 1.37)

Logistic regression models-odds ratio (OR), (confidence intervals 95% CI) and number of women (N).

*p < 0.0001 **p < 0.05 ***p < 0.1

In order to identify if there were socioeconomic disparities in use of mammography within the four population groups of women, logistic regression models were run separately for each group. Table 2 presents logistic regression models explaining mammography-use during 2002 in each group. Education and employment were not associated with mammography-use in any of the groups. Among immigrant women, the OR for having a mammogram indicates that the odds of an unmarried woman having a mammogram were lower than a married woman, however the p value was borderline (p = 0.076). Among Arab women, an OR of less than one may indicate that the odds of younger women having a mammography are higher than that for older women, however again the p value is only borderline (p = 0.085).

**Table 2:** Factors associated with mammography-use during the previous two years in 2002, by population group.

	Non-immigrant, non-ultraorthodox Jewish women N = 273			Ultraorthodox Jewish women N = 247			Immigrants women N = 291			Arabs women N = 246		
	N	OR	(95% CI)	N	OR	(95% CI)	N	OR	(95% CI)	N	OR	(95% CI)

Age	-	0.98	(0.93, 1.03)	-	0.99	(0.93, 1.05)	-	1.01	(0.96, 1.07)	-	0.94*	(0.88, 1.01)

Education												

Academic	92	1.00	ref	52	1.00	ref	188	1.00	ref	29	1.00	ref

High school diploma	93	1.04	(0.58, 1.88)	109	0.97	(0.49, 1.90)	95	0.73	(0.42, 1.28)	36	0.55	(0.18, 1.68)

Less than High school diploma	88	0.77	(0.42, 1.41)	86	1.13	(0.55, 2.30)	8	0.36	(0.04, 3.01)	181	0.60	(0.25, 1.45)

Marital status												

Not married	81	1.00	ref	27	1.00	ref	126	1.00	ref	65	1.00	ref

Married	192	1.26	(0.74, 2.14)	220	1.08	(0.47, 2.48)	165	1.62*	(0.95, 2.75)	181	1.20	(0.58, 2.48)

Employment												

Unemployed	165	1.00	ref	138	1.00	ref	189	1.00	ref	229	1.00	ref

Employed	108	0.91	(0.54, 1.56)	109	1.23	(0.72, 2.11)	102	1.30	(0.71, 2.40)	17	0.38	(0.10, 1.46)

Logistic regression models-odds ratio (OR), confidence intervals (95% CI) and number of women (N).

*p < 0.1

In 2007, no disparities based on age, education, marital status and employment were observed in any of the groups (Table 3).

**Table 3:** Factors associated with mammography-use during the previous two years in 2007, by population group.

	Non-immigrant, non-ultraorthodox Jewish women N = 273			Ultraorthodox Jewish women N = 247			Immigrants women N = 291			Arabs women N = 246		
	N	OR	(95% CI)	N	OR	(95% CI)	N	OR	(95% CI)	N	OR	(95% CI)

**Age**	-	1.03	(0.98, 1.07)	-	0.98	(0.94, 1.03)	-	1.01	(0.67, 1.06)	-	1.01	(0.97, 1.05)

**Education**												

Academic	137	1.00	ref	80	1.00	ref	249	1.00	ref	47	1.00	ref

High school diploma	121	1.21	(0.69, 2.13)	159	0.59	(0.31, 1.12)	112	1.04	(0.63, 1.72)	56	1.57	(0.63, 3.92)

Less than High school diploma	141	0.70	(0.40, 1.23)	108	1.48	(0.70, 3.15)	13	0.70	(0.22, 2.27)	262	0.73	(0.36, 1.49)

**Marital status**												

Not married	110	1.00	ref	35	1.00	ref	149	1.00	ref	88	1.00	ref

Married	289	1.19	(0.72, 2.00)	312	1.41	(0.65, 3.10)	225	1.27	(0.79, 2.03)	277	1.00	(0.59, 1.70)

**Employment**												

Unemployed	205	1.00	ref	184	1.00	ref	208	1.00	ref	323	1.00	ref

Employed	194	0.88	(0.54, 1.46)	163	0.79	(0.47, 1.33)	166	0.93	(0.54, 1.60)	42	1.05	(0.51, 2.20)

Logistic regression models-odds ratio (OR), confidence intervals (95% CI) and number of women (N).

## Discussion

### Disparities between groups

Seven years after the enactment of the National Health Insurance Law in 1995, in which women aged 50–74 were entitled to a biennial mammogram as part of their health insurance plan, less than half the women entitled had a mammogram during the previous two years. The disparities between the non-immigrant, non-ultraorthodox Jewish women and ultraorthodox Jewish women and the immigrants from the fSU and Arabs were large. Age, education, employment and marital status did not explain these disparities and the OR for having a mammogram were less than half among the Arab and immigrant women compared to non-immigrant, non-ultraorthodox Jewish women. These data correspond well with the disparities between non-immigrant non-ultraorthodox Jewish women, immigrant and Arab women measured by self-reported mammography-use in national surveys [25,27]. However, there are discrepancies in the actual percent of women having a mammogram in this study compared to the percent reported in the national surveys. Comparatively, in the self-reported data, women reported higher levels of mammography-use. These differences may be due to the fact that the MHS members are not a representative sample of the Israeli female population or be due to discrepancies between claims data and self-reported mammography-use. Whereas this study is based on claims data, the national surveys are based on self-reported data. The data from this study support the explanation that the differences are due mainly to the bias in self-reporting of mammography-use, as women in this study tend to report having a mammogram more often than is registered in the claims records [28]. In addition, there are also different levels of reliability of self-reported mammography in the different population groups adding to the problematic use of self-reported data, especially when comparing between population groups [28]. The definitions of the population groups in the national studies were similar to the definitions in this study.

The disparities observed in 2002 decreased gradually during the five years of follow-up, until no disparities were observed in 2007 among the Jewish population groups (non-immigrant non-ultraorthodox, ultraorthodox and immigrant women), and only a small difference between Jewish and Arab women was apparent in the crude rates. After adjusting for age, education employment and marital status, the difference between Jewish and Arab women was not highly significant and had only borderline significance. It may be that in a larger sample we would have observed a significant difference between Arabs and Jews. In any case, the disparity between Jews and Arabs during the 5 years decreased considerably. The use of the mobile mammogram may have helped in decreasing the disparities between Jews and Arabs even though its effect on the overall rate of mammography-use was small as the mobile mammogram was sent to peripheral towns and villages serving many Arab women.

The increase in mammography-use was mainly during the years 2005 and 2006; during the year 2007 not much change occurred. Most of the effort MHS put into encouraging women to have a mammogram was implemented since the end of 2004. This may explain the increase during the years 2005–2006. Mammography-use serves as a quality assessment measure and therefore MHS put much effort into increasing levels of mammography-use. The fact that no further increase occurred after 2006 suggests that new strategies and methods should be developed in order to further increase adherence to mammography recommendations. It may also be that it is easier to bring women to have a first time mammogram but harder to bring them in to have a mammogram consistently every two years. At this stage, the type of interventions needed to target the "hard to reach" groups that have not been successfully convinced to have a mammogram during the two years of intervention in which mail and telephone reminders were used are not enough. Interventions going beyond reminders and invitations to mammography are needed.

Although we do not have a control group that did not receive reminders and invitations to have a mammogram, the results may suggest the actions adopted by MHS have been successful in closing the gap between the various groups of Jewish women and Arab women.

Reaching between 67%–74% of mammography-use among women aged 50–74 compares well with other western countries such as the USA where about 72% of women aged 50–74 had a mammogram in 2005 [29]. Other countries have achieved similar results [30]. However, there are countries with higher rates of mammography-use such as Sweden (81%) [31] and Finland (82%) [32] and therefore it is clear that more effort is needed to reach higher goals.

The fact that the differences between Jewish and Arab women in mammography-use have decreased suggests that in the future, there may be a decrease in the differences observed in stage of diagnosis of breast cancer reported previously [4].

### Socioeconomic disparities

In the USA, socioeconomic status (SES) has been shown to be associated with mammography-use; women with less income and lower education received less mammograms and the disparities based on income were greater than the disparities based on education, this persisted during the years [16]. Similar results have been reported in other western countries [33]. However, in this study socioeconomic disparities in mammography-use were not detectable in this sample size. It seems that the services given by MHS are equally distributed and used by women in the different levels of SES. This was true in 2002, and the efforts to increase levels of mammography-use were effective equally in both high and low SES. Therefore, no SES disparities were observed at the end of the 5 year follow up period in the total population in 2007. The significant difference observed in 2002, where the odds of non-married women to receive a mammogram were lower than married women, was non-significant in 2007. This suggests that the efforts of MHS reached both married and non-married women and decreased the previous disparity based on marital status. The observation that non-married women have less mammograms has been reported elsewhere [30]. In addition, the SES disparities in mammography-use, evident in other western countries, are not evident within each population group [16].

All in all, it seems that the actions implemented by MHS were equally successful in all population groups and in all SES levels. There is a need to further increase mammography-use as around 30% of women of the recommended ages still have not had a mammogram in the previous two years.

Other barriers to mammography-use exist in addition to the well studied SES factors such as emotional barriers and beliefs and attitudes [19]. To overcome these barriers and reach a higher level of mammography-use, interventions targeting these beliefs may need to be developed. This would require studies on the beliefs and attitudes of these various groups. It is interesting to point out that the rates of 67%–74% of mammography-use are similar to those reported in other countries. This may suggest that the group of women not yet adhering to the recommendations are the more "hard to reach" for which just invitations and reminders alone will not bring them to have a mammogram; this may be true not only in Israel but also in other societies.

### Strength and limitations

The main strength of this study is the measure of mammography-use based on data from the claims records. A large number of studies use self-reported data, such studies were found to have variations in validity between population groups [6,25-28]. When estimating the success of interventions and comparing various population groups, it is essential that the validity of the outcome will not depend on the population group. Our data enables such a comparison between groups.

This study has a few limitations. The study population is a random sample of only 25% of the Israeli population. It does not represent mammography-use in the whole Israeli society. However, this is less relevant as this study represents achievements that can be reached within a large healthcare organization.

The Arab population in this study does not include women living in mixed cities such as Haifa, as MHS cannot identify them within their database, however, as mentioned, this group is small. Thus, although the data regarding Arab women may not be representative of the total Arab population in Israel, it never the less does not diminish the importance of the results in decreasing the disparities.

The fact that the response rates of each group varied may serve as a limitation. However, we do not expect a differential response bias between women answering the questionnaire and those that were not, as women did not know what the questionnaire was about before refusing and we did not find that the socioeconomic variables were associated with mammography-use. In addition, the trends in mammography-use for each group should not depend on the differential response rates.

Another limitation is the inclusion of all mammograms both screening and non-screening. We do not think this biases the results, as women with breast cancer were not included in the study. In addition, we could not identify women joining MHS during the five years of follow up, and therefore not having a mammogram registered in the claims records. This group is very small.

## Conclusion

During the five years of follow up among members of MHS, the disparities between the population groups in Israel decreased, and no SES disparities are evident. The mammography services provided by the MHS seem to be equally distributed to both high and low SES populations. However, the disparities between Jewish and Arab women have not been completely eliminated and further effort is needed to increase levels of mammography-use among Arab women. There is a need to further increase the percent of women adhering to mammography recommendation to achieve higher rates of mammography-use. These interventions may need to go beyond invitations and reminders in order to reach the more "hard to reach" women.

## Abbreviations

fSU: former Soviet Union; SES: socioeconomic status; MHS: Maccabi Healthcare Service; HMO: Health Maintenance Organization.

## Competing interests

The authors declare that they have no competing interests.

## Authors' contributions

OBE initiated, designed, and run the study, performed the statistical analysis and wrote the manuscript. NF participated in the design of the study, collecting the data and helped draft the paper. OL participated in the designing of the study and helped draft the paper. All authors read and approved the final manuscript.

## Funding

Maccabi Institute for Health Services Research of Maccabi Healthcare Services, Israel.
